# Determinants of Smoking Initiation Among Undergraduate Medical Students in Central India: A Cross-Sectional Study

**DOI:** 10.7759/cureus.104577

**Published:** 2026-03-02

**Authors:** Garima Suryavanshi, Neha Rai, Nandini Goyal

**Affiliations:** 1 Department of Physiology, LN Medical College and Research Center, Bhopal, IND; 2 Department of Anatomy, LN Medical College and Research Center, Bhopal, IND; 3 Department of General Medicine, LN Medical College and Research Center, Bhopal, IND

**Keywords:** cigarette smoking, health professionals, lung, smoking, stress, tobacco, undergraduate medical students

## Abstract

Background: Tobacco use continues to pose a major health challenge in India, and undergraduate medical students form a particularly important group because of their future role in patient care and health promotion. Understanding what influences their smoking habits is crucial for guiding preventive strategies. This study explored the key factors linked to smoking behaviour among undergraduate medical students.

Materials and methods: A cross-sectional questionnaire-based observational study was designed to find determinants of smoking in undergraduate medical students from various colleges in central India. Participation was voluntary, and responses were collected anonymously after obtaining consent.

Results: A total of 489 students responded to the survey. Most participants were male and in their early 20s. Smoking was more common among young male undergraduate students in the age group of 20-22 years. Emotional distress, curiosity, peer interactions, and academic pressure were among the most frequently reported reasons for initiating or continuing smoking. The result showed that family and medical support were significantly related to quitting smoking among undergraduate medical students.

Conclusion: Smoking behaviour among undergraduate medical students appears to be shaped by both demographic characteristics and psychosocial influences. While no single factor independently predicted smoking, family and medical support may be the key factors in helping individuals to quit smoking. Strengthening support systems, stress-management initiatives, and counselling services may help foster healthier, tobacco-free habits among future healthcare professionals.

## Introduction

Tobacco smoking is one of the most important and preventable causes of morbidity and mortality, associated with a variety of diseases such as pulmonary, gastrointestinal, cardiovascular, and various malignant conditions [1,2]. Every year, nearly six million people die worldwide due to tobacco smoking, either directly or indirectly, and non-smokers are exposed to second-hand smoke. So, by 2030, tobacco is estimated to kill more than eight million people across the world per year, if the same trend continues [3-5]. To further add to and worsen the current situation, the majority of smokers start this addictive habit of smoking in their adolescence [5,6].

Health professionals (including undergraduate medical students), paramedical staff, and nurses should ideally not only play an important role in fighting against tobacco use but also serve as role models for society to adopt healthy practises [7]. But on the contrary, several reports suggest that a good number of undergraduate medical students are not only addicted to tobacco but also start very early [8-10]. Hence, this study was conducted to find what made them indulge in this bad habit of smoking despite their knowledge of smoking-related diseases.

## Materials and methods

This was a cross-sectional questionnaire-based observational study conducted in the Department of Physiology, L. N. Medical College, Bhopal, Madhya Pradesh, India, in August and September 2024 to find the determinants of smoking. The study was approved by the institutional ethics committee of L. N. Medical College and Research Centre and J.K.Hospital (reference number: LNMC&RC/Dean/2024/Ethics/075). Students were included from 16 colleges in Madhya Pradesh, India: four colleges each from Bhopal and Indore, two colleges from Nagpur, and one college each from Jabalpur, Gwalior, Shehdol, Bilaspur, Yawatmal, and Raipur.

Study population and study tool

The primary eligibility criterion for participation was that the individual had to be an undergraduate medical student. No specific exclusions based on gender, socio-economic status, or health status were applied, as the study aimed to include a diverse cohort for a comprehensive assessment. The study utilised the WHO Global Adult Tobacco Survey (GATS) questionnaire [11] in Google Forms (Google LLC, Mountain View, California, United States). 

The recruitment drive for the study was started two weeks prior to the commencement of the study, to enrol the maximum number of undergraduate medical students from different medical colleges of Central India. First, a group was created in WhatsApp (Meta Platforms, Inc., Menlo Park, California, United States) comprising undergraduate medical students. An email and WhatsApp message were circulated among them, providing detailed information about the study. The contact numbers and email addresses were got via an Academic Medical Fest organised in Bhopal. The messages included a link to a detailed online consent form and details on how to contact the research team for any queries. Interested students were encouraged to ask questions and clarify doubts before committing to the study. Students who expressed interest in participating and gave informed consent for the study were provided with the GATS questionnaire in Google Forms format. Students were also made aware that their participation was voluntary, their data would be kept confidential, and that they could withdraw from the study at any time without any negative consequences.

Once the consent was given, students were officially enrolled in the study. The recruitment drive successfully enrolled 489 undergraduate medical students. No formal sample size calculation was performed, as we considered the entire undergraduate medical student population in the different colleges.

Data collection and analysis

Responses were collected on an Excel sheet (Microsoft Corporation, Redmond, Washington, United States) and analysed using different statistical methods such as cross tabulation and Chi-square test, using IBM SPSS Statistics for Windows, version 29:0 (Released 2022; IBM Corp., Armonk, New York, United States). p-value < 0.05 was considered significant.

## Results

A total of 489 completed questionnaires were received. Table 1 highlights the demographic distribution of the participants. The majority were male (n= 264, 54.0%) and aged 20-22 years (n= 313, 64.0%). A smaller percentage of students belonged to other age groups, and a few preferred not to disclose their gender.

**Table 1:** Distribution of study participants according to their demographic characteristics (N=489)

Characterisitcs		Frequency	Percentage
Gender	Male	264	54.0
	Female	222	45.4
	Prefer not say	3	0.6
	Total	489	100.0
Age Group (In Years)	17-19	102	20.9
	20-22	313	64.0
	23-25	71	14.5
	26 & above	3	0.6
	Total	489	100.0

A total of 78 participants reported smoking at least once. All the students put in the "non-smokers" group never attempted smoking, while those who smoked at least once were included in the 'Smokers" group, even though they may have quit. Table 2 explains the cross-tabulation of smoking status among the participants versus their demographic characteristics. Several key findings emerged. The data revealed a significant gender difference in smoking prevalence, with a higher proportion of male participants (n= 59, 75.6%) reporting smoking compared to female participants (n= 17, 21.8%) and individuals who preferred not to disclose their gender (n= 2, 2.6%). This difference was statistically significant (p-value < 0.001).

**Table 2:** Cross-tabulation of smoking among the participants versus demographic characteristics *Significant (p-value < 0.05) Chi-square test used.

Characteristics		Smokers (n=78)		Non-Smokers (n=411)		p-value
		Frequency	Percentage	Frequency	Percentage	
Gender	Male	59	75.6	205	49.9	< 0.001*
	Female	17	21.8	205	49.9	
	Prefer not to say	2	2.6	1	0.2	
	Total	78	100.0	411	100.0	
Age group (years)	17-19	6	7.7	96	23.4	0.004*
	20-22	53	67.9	260	63.3	
	23-25	18	23.1	53	12.9	
	26 and above	1	1.3	2	0.5	
	Total	78	100.0	411	100.0	

Regarding age, smoking prevalence was highest among participants aged 20-22 years (n=53, 67.9%), followed by those aged 23-25 years (n= 18 (23.1%), while the oldest age group (26 years and above) had the lowest smoking rate (n= 1, 1.3%). The differences in smoking behavior across age groups were also statistically significant (p-value = 0.004).

When all the predictors were analysed together, several key influences were seen responsible for the initiation of smoking among undergraduate medical students (Table 3). Emotional stress, including family or personal issues, was the most commonly reported factor (n=53, 67.9%), indicating it as a reason for smoking. Curiosity was the second most common factor (n=44, 56.4%), followed by peer pressure (n=34, 43.6%) and academic pressure (n=33, 42.3%). Fitting in with peers and socializing was the factor for 29 participants (37.2%). Less frequently cited reasons included the desire to gain popularity among peers (n=10, 12.8%) and other miscellaneous causes (n=8, 10.3%), indicating they are not dominant motivators.

**Table 3:** Factors responsible for initiation of smoking among participants who smoked (N=78)

Factors responsible to start smoking	Frequency	Percentage
Emotional stress (family or personal issues)	53	67.9
Curiosity	44	56.4
Peer pressure	34	43.6
Academic pressure	33	42.3
Socialising	29	37.2
Gain Popularity amongst peers	10	12.8
Others	8	10.3

Table 4 compares the types of support required for quitting smoking among individuals who smoked (n=78) and those who did not (n=411), along with statistical significance values. Counselling was reported by 43.6% of smokers and 50.1% of non-smokers, with no significant difference (p=0.209), suggesting that counselling is perceived as equally important across both groups. Peer support showed similar proportions (44.9% vs. 46.0%) and was not statistically significant (p=0.856), indicating that peer influence is recognized as a supportive factor regardless of smoking status. Family support, however, showed a significant difference (p=0.010), with only 25.6% of smokers identifying it as necessary compared to 41.1% of non-smokers. Educational support was nearly identical between groups (26.9% vs. 26.8%; p=0.907), showing no meaningful difference. Medical support revealed another significant difference (p=0.006), with fewer smokers (26.9%) reporting it compared to non-smokers (43.6%). 

**Table 4:** Distribution of participants according to types of support reported to be required to quit smoking by the smokers and the non-smokers *Significant (p-value < 0.05); Chi-square test used.

Type of support		Smoking		Total	p-value
		Yes (n=78), n (%)	No (n=411), n (%)		
Counselling	Yes	34 (43.6%)	206 (50.1%)	240	0.209
	No	44 (56.4%)	205 (49.9%)	249	
Peer support	Yes	35 (44.9%)	189 (46.0%)	224	0.856
	No	43 (55.1%)	222 (54.0%)	265	
Family support	Yes	20 (25.6%)	169 (41.1%)	189	0.010*
	No	58 (74.4%)	242 (58.9%)	300	
Educational	Yes	21 (26.9%)	110 (26.8%)	131	0.907
	No	57 (73.1%)	301 (73.2%)	358	
Medical	Yes	21 (26.9%)	179 (43.6%)	200	0.006*
	No	57 (73.1%)	232 (56.4%)	289	

## Discussion

Despite their role as future health providers to the community, a significant number of young undergraduate medical students engage in smoking. This study aimed to find out the factors influencing their behaviours to indulge in smoking and later becoming their habit. We found that cigarette smoking is a real problem among both young male and female undergraduate medical students.

Our study highlights that out of the total 489 participants, the majority were male (54.0%) and aged 20-22 years (64.0%). This demographic distribution suggests that smoking-related behaviors and associated factors are predominantly observed among young adults, particularly those in their early 20s. These findings are consistent with those reported by Al-Kaabba et al. [12].

Cross-tabulation with age and gender revealed a significant difference between genders in smoking prevalence, with a much higher proportion of male participants reporting smoking (75.6%) compared to female participants (21.8%). This observation aligns with findings from previous studies conducted by Al-Kaabba et al. [12], İçmeli et al. [13], and Prijić and Igić [14]. The lower prevalence of smoking among female students may be attributed to sociocultural norms, as smoking is often perceived as socially unacceptable behavior for women. Additionally, underreporting among female participants due to fear of stigma cannot be ruled out [14].

Regarding age, smoking prevalence was highest among participants aged 20-22 years (67.9%). This may reflect exposure to academic stressors, greater social independence, or cumulative behavioral reinforcement over time. These trends have also been documented in studies from Saudi Arabia [11] and Turkey [12]. These findings suggest that smoking prevalence varies by both gender and age group, with male students and younger age groups being more likely to smoke.

The study highlights that smoking habit initiation among medical students is influenced by a range of factors, including emotional stress, curiosity, academic and peer pressure, and social factors. These factors, along with personal knowledge, attitude, and psychological factors, react to create a conducive environment that causes initiation of smoking addiction [15]. Emotional stress (family or personal issues) is the most common cause chosen by the participants to induce smoking behaviour. This is probably because students wrongly believe that smoking is the most convenient coping mechanism to deal with psychological upset, providing stress relief [15,16]. The behavioural influences of curiosity, peer pressure, academic stress, and social interactions were also found to significantly increase the likelihood of initiating smoking [15,17]. These findings are consistent with the observations reported by Akpinar et al. [18] and Çelik et al. [19].

We also found that the undergraduate medical students perceive family support as less accessible or less effective, highlighting a potential gap in family involvement in cessation efforts. Also, smokers underestimate or underutilize medical assistance in quitting, despite its proven effectiveness. Thus, an important intervention for smoking cessation could be a proper, positive, healthy local environment, for example, a smoke-free university campus. For this, mobilising public media, school educators, youth organizations, universities, and others to keep schools and universities smoke-free by the government is important [20].

The findings of our study highlight the importance of addressing smoking behavior among undergraduate medical students as part of professional development and preventive healthcare training. Medical institutions should integrate stress-management programs, mental health support, and structured tobacco-cessation training into medical curricula. From a policy perspective, strengthening smoke-free campus regulations and ensuring access to confidential counseling services may reinforce healthy behaviors and enhance self-motivation for cessation.

Limitations

Though the sample size of 489 undergraduate medical students allowed the estimation of the overall determination of initiation of smoking habit, it limited subgroup analysis. Also, tobacco use was not quantitatively assessed, and psychosocial factors were measured broadly in our study.

## Conclusions

Our study emphasizes the need for integrated tobacco-control strategies in medical colleges, including smoke-free campuses, counseling services, and stress-management programs. Further longitudinal studies are required to better understand causal relationships and to assess the effectiveness of targeted interventions.
